# Application and progress of nomograms in gastric cancer

**DOI:** 10.3389/fmed.2025.1510742

**Published:** 2025-01-29

**Authors:** Haiyu Wang, Yumin Ding, Min Zhuang, Kaixu Li, Shujing Zhao, Dehong Li

**Affiliations:** ^1^School of Public Health, Gansu University of Chinese Medicine, Lanzhou, Gansu, China; ^2^College of Acupuncture-Moxibustion and Tuina, Gansu University of Chinese Medicine, Lanzhou, Gansu, China; ^3^Department of Blood Transfusion, Gansu Provincial Hospital, Lanzhou, Gansu, China

**Keywords:** gastric cancer, nomogram, risk prediction, disease assessment, treatment response, prognosis evaluation

## Abstract

Gastric cancer, as one of the malignant tumors with a significant disease burden globally, emphasizes the importance of early diagnosis and personalized treatment for improving patient prognosis. In recent years, clinical prediction models (CPMs) have played a crucial role in predicting disease risks, assisting medical decision-making, and evaluating clinical prognosis and benefits as tools for risk–benefit assessment. Nomograms, as an important visualization form of clinical prediction models, have been increasingly applied in tumor-related research. Numerous studies have constructed multiple nomogram models by integrating clinical, pathological, laboratory, imaging data, and genetic characteristics, providing an accurate and effective tool for predicting the risk of gastric cancer, early diagnosis, treatment response assessment, and prognosis analysis. This article aims to review the current clinical applications and research progress of nomograms in gastric cancer, with the goal of providing robust references and theoretical support for clinical practice.

## 1 Introduction

Gastric cancer (GC) is prevalent malignant tumors of the digestive system, ranking fifth and fourth in cancer incidence and mortality, respectively. Although a recent decrease in its incidence, GLOBOCAN 2020 statistics indicate that there are still over 1 million new cases of GC and 700,000 deaths worldwide each year (1–3). These data highlight the disease burden of GC globally, especially in East Asia where prevention and control efforts face severe challenges. The early symptoms of GC are usually unobvious and lack specificity, resulting in most patients being diagnosed at an advanced stage, missing the optimal treatment window and leading to poor prognosis (4). Early screening, diagnosis, and personalized treatment plan for GC hold great significance for improving patient prognosis. In addition, traditional prognostic evaluation methods mainly rely on tumor staging systems, often failing to fully consider individual differences among patients (5). Accurate prognostic evaluation is essential for improving treatment decisions and quality of life for GC patients.

In recent years, clinical prediction models (CPMs) have played an important role in predicting disease risks, assisting medical decision-making, and evaluating clinical prognosis and benefits as a tool to assess risks and benefits, becoming an effective way to translate clinical research into clinical practice (6, 7). Nomograms, as a common visualization form of CPMs, have been increasingly applied in tumor-related research. Especially in the field of GC, the application of nomograms in efficacy monitoring and prognostic evaluation has received widespread attention, and there are also related studies exploring its potential value in GC, risk prediction and disease assessment (8–10). This article aims to review the current clinical applications and research progress of nomograms in GC, providing a reference and theoretical basis for clinical practice.

## 2 Methods

This article presents a comprehensive review of relevant English- articles in the PubMed database as of July 16, 2024. The search strategy employed the following terms: (“Gastric cancer” OR “Gastric carcinoma” OR “Cancer of Stomach” OR “Stomach neoplasms” OR “Stomach cancer”) AND (Nomograms OR “Predictive Nomograms”). The review included all research articles, systematic reviews, and meta-analyses. However, case reports, brief communications, editorials, articles in non-English languages, and those with little relevance to the scope of this literature review were excluded.

## 3 Results

### 3.1 Overview of nomograms

The nomogram is a data visualization tool. The basic principle is to build a multi-factor regression model, assign points to each influencing factor according to their contribution to the outcome variable, and then aggregated to get a total score. Finally, a functional transformation relationship between the total score and the probability of the outcome event is obtained. Thus, the predicted value of the outcome event of the individual is calculated, and the prediction model is finally displayed in a graphical way (11, 12). The process of constructing a nomogram generally includes: determining outcome variables and study population, selecting inclusion variables, and constructing a model. In order to evaluate the predictive performance of the nomogram and to judge its applicable population, the model needs to be validated and evaluated. Model validation includes internal validation and external validation. Current studies often evaluate the differentiation, calibration, and clinical utility of the nomogram (13, 14).

### 3.2 Application of nomograms in GC risk prediction

The timing of diagnosis and treatment is closely related to the prognosis of patients with GC. The 5-year survival rate of patients with early GC after active treatment such as surgery is as high as 90%, while the 5-year survival rate of patients with advanced GC is less than 30% (15, 16). Therefore, early identification of high-risk individuals for GC and prompt diagnosis are crucial for improving patient survival and prognosis. However, it is inefficient and difficult to use gastroscopy and pathological biopsy as universal screening tools. In response to this challenge, Wang et al. (17) conducted a multicenter cross-sectional study, retrospectively analyzing clinical data from 7,945 patients in 115 hospitals in China. Within the study, two nomogram models were established based on individual characteristics, laboratory testing indicators, and dietary habits to predict the Operative link for gastritis assessment (OLGA) arbitrary stage and OLGA III-IV stage in high-risk GC populations in China. These nomogram models can predict the presence and severity of gastric atrophy, contributing to the early identification and screening of high-risk individuals for GC and potentially reducing the reliance on endoscopic examinations in clinical practice.

In addition, Sun et al. (18) developed and validated a nomogram model for the diagnosis of gastric low-grade intraepithelial neoplasia (LGIN) in patients with chronic atrophic gastritis (CAG) who underwent gastroscopy biopsy. The model incorporated 10 predictive factors, including age, sex, lesion location, intestinal metaplasia, multiple location, lesion size, erosion, edema, surface white fur, and form, to present the results in a visual nomogram. This nomogram can be used for the diagnosis of high-risk LGIN patients, significantly improving the detection rate of precancerous lesions of GC through enhanced surveillance and active treatment, thereby reducing the incidence of GC. Consequently, this predictive model can be used for individualized prediction of LGIN or as a further supplement to the indications for endoscopic biopsy.

At present, endoscopic forceps biopsy (EFB) is an essential tool for the histopathological diagnosis of early gastric neoplasms (EGN). However, when used in isolation, EFB has limited clinical value in the preoperative assessment of EGN lesions (19, 20). To enhance the accuracy of prediction, Zhao et al. (10) developed a nomogram model based on clinical, laboratory, and endoscopic observation characteristics. This model can objectively and accurately predict the individual risk of pathological upgrading in patients with EGN before undergoing endoscopic submucosal dissection (ESD), demonstrating excellent calibration and discrimination. Another study conducted the first whole-genome analysis of long non-coding RNA (lncRNA) in tissue and plasma samples, identifying five new plasma lncRNAs (TINCR, CCAT2, AOC4P, BANCR, and LINC00857) as potential diagnostic biomarkers for GC. Building upon these lncRNAs, the researchers further developed a nomogram model aimed at promoting its clinical application in diagnosis (21). Table 1 describes nomograms on the prediction of the risk associated with GC. These nomograms can be used to predict the risk grade of GC patients and the individual risk of pathological escalation, enabling early identification and screening of GC while reducing reliance on endoscopy.

**Table 1:** Nomograms on prediction of the risk associated with gastric cancer.

Study	Sample size	Variables standard	Included variables	Outcome measure	Model evaluation	Model validation		
					Discrimination	Calibration	Internal	External
Wang et al. (17)	7,945	demographic data, clinical data, laboratory parameters and dietary habits	OLGA any-stage: Age, PG I, PG II, PG I/II ratio, CEA, HP infection, and white meat; OLGA stages III–IV: age, PG I, PGR, smoking, HP infection, and white meat	predict OLGA any-stage and OLGA stages III–IV	OLGA any-stage:0.610*; OLGA stages III–IV:0.702*	GiViTI calibration belt: OLGA any-stage: *p* = 0.164; OLGA stages III–IV:0.892	OLGA any-stage:0.615*; OLGA stages III–IV: 0.714*	None
Sun et al. (18)	1756	demographic data and clinicopathological characteristics	age, sex, lesion location, intestinal metaplasia, multiple location, lesion size, erosion, edema, surface white fur, and form	diagnose gastric low-grade intraepithelial neoplasia (LGIN)	0.841*	H-L test: *p* = 0.612	0.833*	0.842*
Zhao et al. (10)	978	demographic data, clinical data, laboratory parameters, and endoscopic characteristics	age, history of chronic atrophic gastritis, symptoms of digestive system, blood high density lipoprotein concentration, macroscopic type, pathological diagnosis of EFB, uneven surface, remarkable redness, and lesion size	predict the probability of pathological upgrading	0.804*	NR	None	0.748*
Lin et al. (68)	2,639	demographic data, laboratory parameters, and endoscopic characteristics	age, sex, PG I/II ratio and Kyoto classification scores	predict the risk of GC	0.790*	NR	0.860*	None
Zhang et al. (21)	321	genomic characteristics	five novel plasma lncRNAs (*TINCR*, *CCAT2*, *AOC4P*, *BANCR* and *LINC00857*)	diagnose GC	0.930*	NR	None	0.910*

H-L test, Hosmer-Lemeshow test; NR, not reported; *area under the receiver operating characteristic curve, AUC; #the C-index.

### 3.3 Application of nomograms in preoperative assessment of GC

Lymph node metastasis (LNM), peritoneal dissemination, and other adverse pathological features are significant indicators of GC progression, usually closely associated with poor prognosis (22, 23). Accurate prediction of adverse pathological stages prior to radical resection of GC is of great significance for comprehensively assessing the patient’s condition, formulating individualized treatment plans, and improving patient prognosis. Currently, for GC patients diagnosed with lymph node metastasis, the standard treatment strategy is still total or subtotal gastrectomy combined with systemic D1+/D2 lymph node dissection (24, 25). Therefore, the presence or absence of lymph node metastasis is a key factor that needs comprehensive assessment in early GC patients. Numerous studies (2, 26–33) have identified a series of clinicopathological factors related to LNM in GC patients. By integrating various clinical, pathological, laboratory test results, and imaging characteristics, researchers have constructed multiple nomogram models. These nomogram models can promote individualized preoperative prediction of LNM in GC patients, providing valuable auxiliary tools for clinicians to making treatment decisions, thus improving the overall treatment outcomes and quality of life for GC patients.

Peritoneal Metastasis (PM) represents the most common form of distant metastasis in GC patients, carrying an extremely poor prognosis (34). Once PM occurs in GC patients, it often leads to a significant decline in quality of life and a marked shortening of survival time (35, 36). In clinical practice, there are some limitations in the sensitivity of traditional imaging examinations to detect PM (37). Therefore, it is particularly crucial to identify the presence of PM using more accurate non-invasive methods preoperatively. Chen et al. (38) retrospectively analyzed clinical, pathological, and demographic parameters of 1,112 GC patients, and identified eight independent risk factors for peritoneal dissemination, including age, sex, tumor location, tumor size, signet-ring cell carcinoma (SRCC), T stage, N stage and Borrmann classification IV. A nomogram model was constructed based on these factors to predict peritoneal dissemination, and its predictive efficacy was confirmed through internal and external data validation. On the other hand, Zhao et al. (39) established a novel nomogram model based on serum glycation biomarkers and clinicopathological characteristics. This model also demonstrated good diagnostic performance (AUC: 0.892), achieving individualized assessment of the risk of peritoneal metastasis in GC patients. Consequently, the above two nomogram models hold great value in accurately predicting PM in patients with GC, and can assist clinicians to make more accurate clinical decisions before surgery.

Table 2 delineates the various nomogram models employed to predict LNM, PM, lymphovascular invasion, perineural invasion, and thickening of the distal gastric wall prior to radical gastrectomy. These predictive models frequently incorporated variables such as tumor size, depth of invasion, T stage, and CT parameters, among others. In conclusion, the precise preoperative prediction of GC progression in patients is crucial for formulating the most effective clinical treatment plans, thereby maximizing therapeutic outcomes and enhancing patient prognosis.

**Table 2:** Nomograms on preoperative assessment of gastric cancer.

Study	Sample size	Variables standard	Included variables	Outcome measure	Model evaluation		Model validation	
					Discrimination	Calibration	Internal	External
Li et al. (26)	210	clinical data and radiomics features	tumor thickness, Borrmann classification and the iodine concentration of the primary tumors at the venous phase (ICVP)	the preoperative individualized prediction of lymph node metastasis (LNM) in patients with GC	0.760*	NR	0.793*	None
Yu et al. (27)	5,606	clinicopathological characteristics	tumor size of >2 cm, submucosal invasion, mixed and undifferentiated histologic types, lower tumor location, presence of LVI, and ulceration	preoperatively predict the risk of LNM in patients with EGC	0.768*	NR	0.760*	None
Pan et al. (28)	1911	endoscopic characteristics and pathological information	tumor size, grade, and T stage	accurately predicts LN metastasis risk for elderly patients with EGC before endoscopic resection	0.723*	NR	0.706*	None
Wang et al. (29)	307	clinical data and pathological characteristics	tumor budding grade, lymphovascular invasion, depth of tumor invasion, ulceration, and tumor differentiation	predicting the status of lymph node involvement in EGC patients	0.872*	H-L test: *p* = 0.834	NR	0.885*
Wang et al. (30)	2,789	radiomics features and clinicopathological characteristics	primary site, T-stage, NLNE, and tumor size	prognosticate LNM in patients with gastric signet ring cell carcinoma (GSRC)	0.798*	NR	0.797*	0.826*
Wu et al. (31)	1,061	laboratory parameters and clinicopathological characteristics	depth of invasion, tumor size, degree of differentiation, and platelet-to-lymphocyte ratio (PLR)	predict lymph node metastasis in patients with EGC	0.775*	H-L test: *p* = 0.684	0.792*	None
You et al. (2)	183	clinicopathological characteristics	tumor size, invasion depth, positive mismatch repair function deficit (dMMR), and macroscopic type	predict lymph node metastasis in early gastric signet ring cell carcinoma	0.757*	NR	NR	None
Yoo et al. (32)	4,482	clinicopathological characteristics	tumor size, tumor depth, cross-sectional location, differentiation, lymphovascular invasion	Predicting extraperigastric lymph node metastasis in patients with EGC	NR	NR	NR	NR
Jiang et al. (33)	2,217	demographic data and clinicopathological characteristics	Age at diagnosis, histology type, grade, T-stage, and tumor size	predict LNM of patients with EGC	0.751*	NR	0.786*	None
Chen et al. (38)	1,112	demographic data and clinicopathological characteristics	age, sex, tumor location, tumor size, signet-ring cell carcinoma (SRCC), T stage, N stage and Borrmann classification IV (Borrmann IV)	predict peritoneal dissemination in GC patients	0.791*	NR	None	0.779*
Zhao et al. (39)	129	clinical data, laboratory parameters and genomic characteristics	Weight loss, CA19-9, CA125, lymphocyte count, H5N5F1E2	predict peritoneal metastasis in GC patients	0.892*	NR	None	None
Tong et al. (69)	171	clinical data, laboratory parameters and radiomics features	Borrmann classification, CA724, tumor thickness, and iodine concentration in the venous phase (VIC)	predict preoperative lymphovascular invasion (LVI) in GC	0.864*	H-L test: good	0.964*	0.877*
Ge et al. (70)	256	radiomics features	CT-T stage, CT-EMVI, VP-70 keV CT value, and EP-NIC	preoperatively predict lymphovascular and perineural invasive risk in GC patients	0.918*	H-L test: *p* = 0.605	0.874*	None
Cong et al. (71)	351	radiomics features, endoscopic characteristics and laboratory parameters	Extramural vascular invasion (EMVI), Borrmann classification, tumor thickness, and the systemic inflammation response index (SIRI)	preoperatively predict perineural invasion (PNI) in advanced GC	0.838*	H-L test: *p* = 0.115	NR	None
He et al. (72)	291	radiomics features	In the LVI group: CT_N stage, RadScore; In the PNI group: clinical stage, RadScore	preoperatively predict lymphovascular invasion (LVI) and perineural invasion (PNI) in GC	In the LVI group:0.792*; In the PNI group: 0.834*	H-L test: *p* = 0.945	In the LVI group: 0.822*; In the PNI group: 0.828*	None
Feng et al. (73)	208	clinical and radiological data	the venous phase spectral curve, focal enhancement, arterial phase mixed, tumor site, and diphasic shape change	estimate the malignant probability of distal gastric wall thickening	0.803*	H-L test: *p* = 0.258	0.905*	None
Chen et al. (74)	718	endoscopic characteristics and clinicopathological characteristics	location, macroscopic type, length, marked margin elevation, WLI color difference and histological type	predict submucosal invasion in EGC	0.881*	None	0.840*	None
Li et al. (75)	1969	demographic data, clinicopathological characteristics and laboratory parameters	Age, Lauren type, signet-ring cell, N stage, immunohistochemical ER expression, serum CA125 and NLR	predict the risk of ovarian metastasis in GC	0.867*	H-L test: good	None	None
Hu et al. (76)	123	clinicopathological characteristics and radiomics features	the ECV fraction, tumor location, and Borrmann type	predict microsatellite instability status in GC	0.826*	H-L test: *p* = 0.146	0.833*	None
Pan et al. (77)	315	radiomics features and laboratory parameters	the radiologic tumor invasion score, PLR, and preoperative hemoglobin	Preoperatively predict serosal invasion of GC r	0.884*	H-L test: *p* = 0.466	0.837*	None
Liu et al. (78)	1,281	laboratory parameters	d-dimer, CA199, CA125, the neutrophil to lymphocyte ratio (NLR) and prognostic nutritional index (PNI)	predict distant metastasis in GC	0.838*	H-L test: good	0.811*	None

H-L test, Hosmer-Lemeshow test; NR, not reported; *area under the receiver operating characteristic curve, AUC.

### 3.4 Application of nomograms in predicting treatment response of GC

Neoadjuvant chemotherapy (NAC) has been demonstrated to effectively reduce tumor burden, downstage the disease, enhance rates of surgical resection, and improve the prognosis of patients with GC (40, 41). Numerous international guidelines recommend NAC as a critical therapeutic approach to improve the therapeutic effect of patients with advanced GC (42, 43). However, the survival benefit conferred by NAC depends on the pathological response to chemotherapeutic agents (44), and there exists considerable inter-individual variability in patient responses to NAC (45). Patients who achieve a complete pathological response often exhibit longer overall survival (OS) and disease-free survival (44), whereas those with poor responses to NAC may face a worse prognosis (46). Therefore, the early and accurate prediction of a GC patient’s response to NAC is of paramount importance for avoiding ineffective treatments and devising personalized therapeutic strategies.

Table 3 describes the various nomogram models used to predict the response to NAC in patients with advanced GC. In recent years, nomograms constructed based on imaging characteristics have emerged as important tools to evaluate the treatment response in GC patients, and provide significant guidance for devising personalized treatment plans. These studies utilized imaging techniques such as computed tomography (CT) and magnetic resonance imaging (MRI), combined with artificial intelligence (AI) technologies like machine learning and deep learning, to construct a series of nomogram models. These nomogram models not only incorporate imaging characteristics but also integrate clinical data, making the predictive models more comprehensive and accurate. The application of AI enhances precision and enables extraction of complex features for constructing nomograms. This approach enables a more precise assessment of patients’ treatment response, providing clinicians with more reliable decision support.

**Table 3:** Nomograms on prediction of the response to NAC in patients with advanced gastric cancer.

Study	Sample size	Variables standard	Included variables	Outcome measure	Model evaluation		Model validation	
					Discrimination	Calibration	Internal	External
Chen et al. (79)	128	radiomics features and laboratory parameters	radiomics score, PLR, ALT/AST, total bilirubin, and CA19-9 levels	Predict therapeutic response to neoadjuvant chemotherapy in locally advanced GC	0.800*	None	0.400*	None
Liu et al. (80)	230	clinicopathological characteristics, radiomics features and laboratory parameters	tumor location, histological differentiation, clinical T stage, and carbohydrate antigen 724	Predict the response to neoadjuvant chemotherapy in patients with advanced GC	0.806*	H-L test: good	None	None
Zhang et al. (81)	322	radiomics features, handcrafted and deep learning features	handcrafted signature, deep learning signature and CT stage	predict neoadjuvant chemotherapy response in locally advanced GC patients	0.848*	H-L test: *p* = 0.054	0.802*	0.751*
Cui et al. (82)	719	radiomics features, handcrafted and deep learning features	the handcraft-based signature, DL-based signature, and CT stages	predict the response to neoadjuvant chemotherapy in patients with locally advanced GC	0.848*	H-L test: *P* = 0.834	0.829*	cohort 1: 0.804*; cohort 2: 0.827
Li et al. (83)	141	clinicopathological characteristics and radiomics features	ADC Radscore, DCE Radscore, T2WI Radscore and Borrmann classification	predict pathological response to neoadjuvant chemotherapy in locally advanced GC	0.844*	H-L test: ood	0.820*	None
Chen et al. (8)	208	pathological characteristics and laboratory parameters	carcinoembryonic antigen level, lymphocyte ratio, monocyte count and tumor differentiation grade	predict pathological complete response to neoadjuvant chemotherapy in patients with advanced GC	0.823*	H-L test: ood	NR	None
Zhong et al. (84)	98	radiomics features and laboratory parameters	cycle number of NACT, delta longest diameter, and post-CA199	predict the response of metastatic lymph nodes to neoadjuvant chemotherapy in locally advanced GC	NR	None	0.940*	None
Li et al. (85)	222	radiomics features and clinicopathological characteristics	Borrmann classification, ICDP, and nICDP	predict pathologic response to neoadjuvant chemotherapy in locally advanced GC	0.797*	NR	0.741*	None

H-L test, Hosmer-Lemeshow test; NR, not reported; *area under the receiver operating characteristic curve, AUC.

In summary, nomograms based on imaging characteristics and AI technologies hold broad application prospects in predicting the response to neoadjuvant chemotherapy in GC and in aiding therapeutic decision-making. Subsequent studies can further optimize nomogram models, take full advantage of AI technologies, integrate a variety of imaging techniques and clinical data, and conduct more external validations with the aim of achieving more precise predictions of treatment responses, thereby providing stronger support for personalized treatment of GC patients.

### 3.5 Application of nomograms in predicting prognosis of GC

#### 3.5.1 Prediction of postoperative complications in GC

In the field of GC treatment, despite significant progress in recent years, radical gastrectomy remains the principal modality of treatment for GC (47). However, postoperative complications not only escalate the cost and duration of hospitalization but also may adversely affect patient prognosis (48, 49). Therefore, the early prediction and identification of the risk of postoperative complications is of great significance for guiding clinical treatment, reducing complications incidence and mortality rates, and improving the quality of life for patients. In the field of predicting postoperative complications in GC, the nomograms have been applied and developed in numerous studies as efficient risk assessment tools. Table 4 describes in detail various models used to predict the risk of complications following radical treatment for GC.

**Table 4:** Nomograms on prediction of complications following radical treatment for gastric cancer.

Study	Sample size	Variables standard	Included variables	Outcome measure	Model evaluation		Model validation	
					Discrimination	Calibration	Internal	External
Tan et al. (86)	101	clinical data and radiomics features	the visceral fat area (VFA), the slope of spectral curve (λ) in venous phase (λ-VP) and tumor Hounsfield units on mon-oenergetic images 40 keV in VP (MonoE_40keV-VP_)	predict postoperative complications (POCs) in patients with GC	0.890*	NR	None	None
HWANG et al. (50)	237	demographic data, clinical data and laboratory parameters	age, approach, operation time, WBC count, NLR and CRP	Predict infectious complications following curative gastrectomy	NR	NR	None	None
Ma et al. (87)	404	demographic data, clinical data and laboratory parameters	age, PNI, PLR, CA199 level, ASA score, and ICU treatment	predict postoperative pulmonary infection following D2 radical gastrectomy for GC	0.736*	H-L test: ood	0.707*	None
Yu et al. (51)	322	clinical data and laboratory parameters	body mass index, glucose, hemoglobin, albumin, surgical duration, and bleeding volume	predict intraabdominal infection after radical gastrectomy in elderly patients	0.933*	H-L test: ood	0.951*	None
Zhou et al. (88)	2,124	demographic data, clinical data and laboratory parameters	age, total cholesterol, total gastrectomy, duration of surgery, and the dose of oxycodone	predict the risk of PPCs in GC patients after elective gastrectomy	0.735*	H-L test: ood	0.781*	None
Zhang et al. (89)	131	clinical data	hypertension, diabetes, history of abdominal surgery, and perioperative blood transfusion	predict early complications after distal gastrectomy	0.843*	H-L test: = 0.501	0.877*	None
Shi et al. (52)	326	clinical data	Anastomotic Score system on postoperative Day 3, tumor location, surgical procedures, and anastomotic type	detect anastomotic leakage after GC surgery in the early phase	0.930*	H-L test: good	0.900*	0.820*
Xu et al. (90)	476	dietary habits and clinical data	smoking history, BMI, anastomosis type, blood loss, and distance from tumor to superior margin	Predicting esophagojejunal anastomotic leakage in GC patients after total gastrectomy	0.956*	H-L test: good	0.947*	None
Zhou et al. (53)	693	demographic data and laboratory parameters	age, D-dimer (D-D) level, low-density lipoprotein, CA125, and calcium and chloride ion levels	Predict lower extreme deep vein thrombosis following radical gastrectomy for GC	0.936*	H-L test: *p* > 0.05	0.875*	None
Zhou et al. (91)	3,092	demographic data, clinical data and laboratory parameters	age, Karnofsky Performance Status (KPS), blood transfusion, Clinical stage, central venous catheterization, operation, fibrinogen degradation product and D-dimer	predict the risk of the appearance of VTE in GC patients	0.820*	H-L test: *p* = 0.863	0.850*	None
Liu et al. (54)	280	demographic data and clinical data	age, American Society of Anesthesiologists (ASA) classification, anesthetic drug consumption, extubation time, and Post-anesthesia Care Unit (PACU) stay	visually predict the occurrence of hyperactive delirium after laparoscopic radical gastrectomy under general anesthesia in patients with GC	0.903*	NR	None	None
Yong et al. (55)	312	demographic data, clinical data and laboratory parameters	age, nutritional risk screening 2002 (NRS2002) score, neutrophil-to-lymphocyte ratio (NLR), albumin-to-fibrinogen ratio and prognostic nutritional index (PNI)	predict Delayed neurocognitive recovery (DNR) in elderly GC patients after radical gastrectomy	0.863*	NR	None	None
Yu et al. (92)	173	demographic data, laboratory parameters and clinicopathological characteristics	Age, WBC count, tumor size, postoperative metastasis, and the interval from gastrectomy to first SBO	predict the recurrence of small bowel obstruction (SBO) after gastrectomy in patients with GC	0.869*	H-L test: good	NR	None

H-L test, Hosmer-Lemeshow test; NR, not reported; *area under the receiver operating characteristic curve, AUC.

Postoperative infection is one of the common complications in GC patients. HWANG et al. retrospectively collected clinical and pathological data from 237 GC patients who underwent radical gastrectomy, and used logistic regression analysis to construct a nomogram model to predict the occurrence of infection after radical treatment for GC. This model incorporates clinical and laboratory parameters such as age, approach, operation time, WBC count, NLR, and CRP (50). Using this model before discharge can assist in identifying individuals requiring additional treatment, thereby minimizing the risk of patient readmission. Furthermore, a retrospective study confirmed that body mass index, glucose, hemoglobin, albumin, surgical duration, and bleeding volume were independent risk factors for intra-abdominal infection, and the nomogram model constructed based on these factors demonstrated excellent predictive performance in both the training set (AUC = 0.933) and the validation set (AUC = 0.951) (51). This model can effectively screen high-risk patients with postoperative intraperitoneal infection and guide clinicians in optimizing perioperative management for such individuals to reduce the incidence of postoperative infection. It is worth noting that the model was established based on an elderly patient population, which reflects the applicability and importance of nomograms in different patient groups. Selection bias is inevitable in these retrospective studies, and it is necessary to externally validate the model in the future to improve their clinical applicability.

Nomograms have also demonstrated high accuracy and practicality in predicting specific complication risks, such as anastomotic leakage (AL), lower extremity deep vein thrombosis (DVT), and hyperactive delirium. Shi et al. conducted the first prospective cohort study with independent internal validation and constructed a nomogram model for early diagnosis of anastomotic leakage after radical treatment for GC by analyzing inflammatory factors in abdominal drainage fluid. The model showed a C-index of 0.93 in the train cohort and a C-index of 0.82 in the validation cohort, demonstrating good predictive power (52). Postoperative venous thromboembolic events, such as lower extremity deep vein thrombosis (DVT), are major risk factors for GC patients after radical gastrectomy. Accurate prediction and management of these risks is essential is critical to improving postoperative care and patient outcomes for GC patients. Zhou et al. (53) developed a nomogram model to predict DVT in the lower extremities after radical gastrectomy for GC, based on various machine learning methods. The model exhibited high predictive value in both the training set (AUC = 0.936) and the validation set (AUC = 0.875), becoming an important tool for clinicians to identify and manage the risk of lower extremity DVT in this patient population. Additionally, nomograms are helpful in predicting hyperactive delirium after laparoscopic radical gastrectomy in GC patients (54) and delayed postoperative neurocognitive recovery in elderly GC patients (55).

In summary, nomograms have broad application prospects in predicting complications after GC treatment. By integrating clinical, laboratory, and imaging parameters, nomograms can provide personalized risk assessment for clinicians, thereby optimizing treatment decisions and patient management. However, these nomograms still need to be validated for their effectiveness and universality through larger sample sizes and further prospective studies.

#### 3.5.2 Prediction of postoperative recurrence in GC

The recurrence of GC after treatment significantly impacts prognosis. In recent years, nomograms have been widely used as effective predictive tools to assess the risk of recurrence in patients with GC. Endoscopic mucosal dissection (ESD) is an effective treatment for early gastric cancer (EGC) (56), and accurately predicting postoperative local recurrence risk is particularly important. Through in-depth analysis, Li et al. (57) identified multiple risk factors for recurrence after ESD in patients with EGC, including diabetes, alcohol consumption, lymphatic infiltration, complications, and multiple lesions. Based on these factors, the study established the first high-precision nomogram model for predicting the risk of EGC recurrence after ESD. In addition, another study developed a nomogram model with higher predictive efficiency (AUC = 0.933) based on the two predictors of lymph node positivity and *helicobacter pylori* (HP) infection (58). These models provide important reference for clinicians to identify high-risk patient groups and plan more reasonable follow-up strategies, as well as reliable data support for the prevention and treatment of postoperative recurrence.

Early detection of recurrence after radical gastrectomy is essential to improve the prognosis and survival rate of GC patients. Huang et al. developed a practical GC recurrence risk prediction model based on Lasso-Cox regression analysis. This model incorporates seven variables, including pathological stage, tumor size, the number of total lymph nodes, the number of metastatic lymph nodes, intraoperative blood loss (IBL), and levels of AFP and CA199, enabling rapid assessment of patients’ risk of recurrence (59). Subsequently, Cao et al. (60) further integrated clinical and pathological characteristics of GC patients to establish a risk model for predicting early postoperative recurrence. This nomogram not only accurately predicts the early postoperative recurrence rate in GC patients and identifies high-risk individuals for postoperative GC recurrence but also provides important guidance for clinicians to formulate appropriate treatment plans and effective follow-up strategies.

Table 5 outlines the various nomogram models utilized to predict postoperative recurrence in patients with GC. In summary, multiple nomograms constructed by integrating clinicopathological features, imaging features and biomarkers of patients is of great significance in predicting postoperative recurrence of patients with GC. These models not only improve the accuracy of clinical decision-making, but also provide a scientific basis for personalized treatment of patients.

**Table 5:** Nomograms on prediction of postoperative recurrence in patients with gastric cancer.

Study	Sample size	Variables standard	Included variables	Outcome measure	Model evaluation		Model validation	
					Discrimination	Calibration	Internal	External
Li et al. (57)	238	dietary habits, endoscopic and clinicopathological characteristics	diabetes, alcohol consumption, lymphatic infiltration, complications, and multiple lesions	predict local recurrence in adult patients with early GC after endoscopic submucosal dissection	0.843*	H-L test: good	NR	None
Xu et al. (58)	408	clinical data	*Helicobacter pylori* and number of positive lymph nodes	predict the post-endoscopic submucosal dissection (ESD) recurrence risk of EGC	0.933*	H-L test: good	NR	None
Huang et al. (59)	649	clinical data, laboratory parameters and clinicopathological characteristics	pathological stage, tumor size, the number of total lymph nodes, the number of metastatic lymph nodes, intraoperative blood loss (IBL), the level of AFP and CA199	predict recurrence risk for GC patients after radical gastrectomy	0.840*	NR	0.756*	None
Cao et al. (60)	521	demographic data, clinicopathological characteristics and laboratory parameters	age, serous infiltration, lymph node metastasis, recurrence mode, and CA19-9	predict early recurrence after radical gastrectomy of GC	0.739*	H-L test: ood	0.743*	None
Huang et al. (93)	366	clinicopathological characteristics	surgical margin, postoperative tumor node metastasis (pTNM) stage, and neural invasion	evaluate the risk of postoperative anastomotic recurrence in patients with Siewert II or III adenocarcinoma who did not receive neoadjuvant therapy	0.750*	H-L test: ood	NR	0.807*

H-L test, Hosmer-Lemeshow test; NR, not reported; *area under the receiver operating characteristic curve, AUC.

#### 3.5.3 Prediction of survival time in patients with GC

Accurate prediction of patient survival is crucial for optimizing treatment strategies and improving prognosis in the field of GC treatment. As efficient personalized prediction tools, nomograms have been widely used for survival prediction in GC patients, and have been continuously improved and optimized in research. Wang et al. (61) developed a nomogram based on inflammation, nutritional and pathological factors to predict the overall survival of patients with GC after radical gastrectomy. In addition, a retrospective dual-center cohort study (62) conducted an in-depth analysis of clinical data of stage II/III GC patients undergoing radical resection and perioperative adjuvant chemotherapy (PAC) at two tertiary hospitals. Based on multivariate Cox regression analysis of the training group, the investigators constructed a nomogram integrating four independent predictors: BMI, total gastrectomy, TNM stage and peri-operative adjuvant chemotherapy (PAC) to estimate the survival probability of patients with GC. The AUC and calibration curve of this nomogram indicated good discrimination and calibration performance. The results of decision curve analysis (DCA) further confirmed that the model has better net benefit compared to the eighth TNM staging system. This nomogram provides a tool for clinicians to assess the probability of survival in GC patients receiving incomplete PAC and helps encourage patients to complete established chemotherapy regimens.

For the special subtype of gastric signed-ring cell carcinoma (GSRCC), several studies have developed nomograms to provide accurate assessment tools for predicting the survival probability of this patients. Jiang et al. (63) developed and verified a nomogram model for predicting overall survival (OS) and cancer-specific survival (CSS) of GSRCC patients by including six variables, such as age, race, tumor size, tumor site, N stage and AJCC stage. The study also created a dynamic web application based on the nomogram to facilitate clinical decision-making. Shao et al. (64) developed another nomogram based on age, TNM staging system, surgery, and chemotherapy as four independent prognostic factors. Compared with the traditional AJCC staging system, the constructed nomograms demonstrated more significant net clinical benefit and predictive value. It is evident that nomograms play an important role in the survival prediction of GSRCC patients. Not only does they provide clinicians with an accurate tool for predicting survival rates for individual patients, they also help develop more personalized treatment strategies.

Furthermore, the role of specific biomarkers or genetic markers in predicting the prognosis of GC has also attracted the attention of researchers (9, 65, 66). For example, a nomogram based on nine differentially expressed genes associated with gut Microflora was developed to predict prognosis and overall survival time in patients with GC (65). The nomogram constructed by Zhong et al. based on five T-cell marker genes and clinical factors could accurately predict the possibility of survival at 1, 3, and 5 years after diagnosis in GC patients (66). The introduction of machine learning technology provides new technical support for the establishment of nomogram models and further improves the accuracy of prediction (67).

Table 6 describes the various nomogram models used to predict survival in patients with GC. To sum up, nomograms play a key role in predicting the survival of GC patients. By integrating multiple factors such as inflammation, nutrition, pathology, tumor markers and genes, nomograms provide more accurate prediction tools for prognosis assessment of patients with GC. Future studies need to further explore and integrate more biomarkers and clinical data to continuously verify and improve the accuracy and clinical practicability of the model, so as to provide more scientific guidance for the treatment and management of GC patients.

**Table 6:** Nomograms on prediction of survival in patients with gastric cancer.

Study	Sample size	Variables standard	Included variables	Outcome measure	Model evaluation		Model validation	
					Discrimination	Calibration	Internal	External
Wang et al. (61)	238	laboratory parameters and clinicopathological characteristics	Age, CA50, prognostic nutritional index (PNI), systemic immune-inflammation index (SII), T stage, and N stage	predict the overall survival (OS) of patients with advanced GC after curative gastrectomy	the AUC for the 1-year, 3- year, and 5- year OS was 0.740, 0.832 and 0.848, respectively	H-L test: *p* = 7.50	0.790*, 0.814* and 0.799*, respectively	None
Liu et al. (62)	1,070	clinicopathological data and operative variables	BMI, total gastrectomy, TNM stage and peri-operative adjuvant chemotherapy (PAC)	predict the probability of survival of patients with stage II/III GC who received incomplete PAC	the AUC values to predict the 1–3-, and 5-year survival probabilities were 0.729, 0.749, and 0.768, respectively	H-L test: good	0.717*, 0.734* and 0.742*, respectively	None
Wang et al. (94)	1879	clinicopathological data	grade, histology, M stage, radiotherapy, tumor size, and T stage	predict cancer-specific survival of elderly patients with unresected GC who received chemotherapy	the AUC for the 3-, 4-, and 5-years CSS were 0.689, 0.708, and 0.731, respectively	H-L test: good	0.666*, 0.693*, and 0.708*	None
Shao et al. (64)	1804	demographic data and clinicopathological characteristics	age, tumor lymph node metastasis (TNM) staging system, surgery, and chemotherapy	predict overall survival (OS) and cancer-specific survival (CSS) in patients with gastric signet ring cell carcinoma (GSRCC)	The AUCs for the 2- and 5-year OS were 0.848 and 0.885, respectively, and those for CSS were 0.854 and 0.899, respectively	NR	NR	NR
Jiang et al. (63)	4,198	demographic data and clinicopathological characteristics	age, race, tumor site, tumor size, N stage, and AJCC stage	assess the overall survival (OS) and cancer-specific survival (CSS) of patients with GSRCC	the 1-, 3-, and 5-year AUC values for OS were 0.76, 0.82, and 0.81, respectively, and those for CSS were 0.76, 0.82, and 0.83, respectively	H-L test: *p* > 0.05	The AUC values were consistently above 0.70	None
Zuo et al. (95)	342	laboratory parameters and clinicopathological characteristics	CA19-9, VFI level, N2 and N3	predict the survival outcomes of patients with GC after surgery	The AUC values for 1-, 3- and 5-year OS were 0.718, 0.691 and 0.731	H-L test: good	None	None
Chen et al. (96)	1,652	clinicopathological characteristics and operative variables	liver metastasis, bone metastasis, primary site, surgery, regional surgery, treatment sequence, chemotherapy, radiotherapy, positive lymph node count, N staging, and time from diagnosis to treatment	predict the 1-year and 3-year survival rates for patients diagnosed with GC with lung metastasis (GCLM)	The AUC values for 1- and 3-year OS were 0.814 and 0.772	H-L test: good	0.687* and 0.602*	None
Meng et al. (97)	5,451	demographic data and clinicopathological characteristics	age, histological type, grade, tumor size, surgery, chemotherapy, bone metastasis, and lung metastasis	predict overall survival and cancer-specific survival in GC patients with liver metastases	The AUC values for 1-, 2- and 3-year OS were 0.788, 0.795 and 0.818; and those for CSS were 0.785, 0.792 and 0.809, respectively	H-L test: good	1-, 2- and 3-year OS:0.801*, 0.803* and 0.824*; 1-, 2- and 3-year CSS: 0.807*, 0.802* and 0.839*	1-, 2- and 3-year OS:0.624*, 0.559*and 0.629*; 1-, 2- and 3-year CSS: 0.608*, 0.557*and 0.634*
Yue et al. (65)	100	genomic characteristics	the risk score model(*HSD17B3*, *GNG7*, *CHAD*, *ARHGAP8*, *NOX1*, *YY2*, *GOLGA8A*, *DNASE1L3*, and *ABCA8*) and Pathologic M	predict the survival in GC patients	The C-index for 1-, 3- and 5-year OS were 0.824, 0.772, and 0.735	NR	None	None
Wei et al. (3)	60	radiomics features and pathological characteristics	ΔBMD, ΔPMA, HER2and maximal tumor diameter	predict disease-free survival(DFS) after surgery and adjuvant chemotherapy in patients with GC	The AUC values for 2- and 3-year DFS were 0.879 and 0.928	H-L test: good	None	None
Huang et al. (98)	294	radiomics features and clinical data	radiomics score (RS) and distant metastasis	predict immunotherapy-related progression-free survival (irPFS)	0.778#	H-L test: good	0.767#	C-Index of the cohort 1: 0.713; C-Index of the cohort 2: 0.687
Deng et al. (99)	124	radiomics features and laboratory parameters	prognostic nutritional index (PNI)-skeletal muscle index (SMI) and Eosi	predict the progression-free survival (PFS) and OS of patients with GC treated with immune checkpoint inhibitor	NR	NR	None	None
Liu et al. (100)	146	laboratory parameters and clinicopathological characteristics	CA724, Geriatric Nutritional Risk Index (GNRI), and TNM stage	predict the prognosis of GC patients treated with immune checkpoint inhibitors	C-Index of PFS: 0.667; C-Index of OS: 0.685	H-L test: good	None	None
Li et al. (101)	760	demographic data, laboratory parameters and clinicopathological characteristics	age, T stage, N stage, radical resection, and Prealbumin Ratio (FPR)	predict the five-year OS of patients with resectable GC	0.859*	NR	None	None
Tian et al. (102)	489	laboratory parameters and clinicopathological characteristics	the OS prediction model: grade, TNM-stage, chemotherapy, and Fibrinogen and Platelet to Pre-albumin Ratio(FPAR);the RFS prediction model: grade, N-stage, TNM-stage, and FPAR	predict the prognosis of patients with advanced gastric cancer(AGC)	the 2- and 3-year AUC of the OS model were 0.737and 0.756; the 2- and 3-year AUC of the RFS model were 0.738 and 0.758	H-L test: good	None	None
Zhang et al. (103)	1,140	laboratory parameters and clinicopathological characteristics	TNM, ALI, AGR, neutrophil-to-lymphocyte ratio (NLR), and PNI	predict the OS of GC patients	The AUC values for 1-, 3- and 5- years OS were 0.753, 0.774, 0.755 at	H-L test: good	None	None
Sun et al. (104)	1,560	demographic data, genomic and clinicopathological characteristics	NK cell-associated signature (NKCAS), age, M stage, and tumor grade	predict the survival outcomes of GC patients	The AUC values of the nomogram at 1-, 3-, and 5-years were 0.763, 0.858, and 0.847	NR	None	None
Lu et al. (67)	404	demographic data, laboratory parameters, pathological characteristics and operative variables	age, gender, lymphocyte count, maximum tumor diameter, CEA level, nerve or vascular invasion, TNM stage, and gastrectomy method	evaluate the prognosis of GC patients who have undergone radical gastrectomy	NR	NR	None	None
Ba et al. (105)	291	clinicopathological characteristics and laboratory parameters	tumor-node-metastasis (TNM) stage, Borrmann type, and prognostic immunoinflammatory index (PII) score	predict prognosis in patients with GC undergoing surgical treatment	the 1-, 3-, and 5-year AUC values of the nomogram for PFS were 0.834, 0.841, and 0.863; those for OS were 0.830, 0.821, and 0.850	NR	None	None
Gao et al. (106)	2,110	demographic data, clinicopathological characteristics and laboratory parameters	age, sex, BMI, LVI, location, CEA, TNM stage and CA199	estimate the net survival gain attributable to the receipt of adjuvant chemotherapy for patients with stage IB GC	The C-indexes for OS were 0.74 in the group treated with adjuvant chemotherapy and 0.70 in the group treated with surgery only	H-L test: good	None	None
Dong et al. (107)	197	laboratory parameters and pathological characteristics	systemic immune-inflammatory index (SII), NLR, platelet to lymphocyte ratio (PLR), and N stage	predict survival outcomes in advanced GC patients undergoing ICIs combined with chemotherapy	The AUC of the nomogram for predicting the 6-, 12-, and 18-month OS were 0.651, 0.745, and 0.771, respectively	H-L test: good	None	0.601*, 0.647*, and 0.808*
Zhong et al. (66)	322	demographic data and genomic characteristics	Age, Gender and TCMG-score(MMP2, SERPINE1、CXCR4, CTLA4 and CXCL3)	predict the prognosis in GC patients	the 1-, 3-, and 5-year AUC values of the nomogram for OS were 0.667, 0.73 and 0.818	H-L test: good	None	None
Maimaiti et al. (108)	21,757	demographic data and clinicopathological characteristics	age, marital status, race, tumor location, pathological grade, histological type, T and N stage, surgery for the primary tumor, radiotherapy, chemotherapy, tumor size, and RNE	predict OS and cancer-specific survival (CSS) for locally advanced gastric cancer (LAGC)	The 2-, 3-, and 5-year AUC values of the nomogram for OS were 76.81, 76.74, and 76.97%; those for CSS were 77.57, 77.87 and 78.13%	H-L test: good	2-, 3-, and 5-year OS:76.18,76.27 and 76.05%; 2-, 3-, and 5-year CSS: 77.16, 76.98 and 77.67%	None
Li et al. (9)	407	demographic data, genomic and clinicopathological characteristics	risk score(*MATN3, ATP2A1, NOX4, AQP11, HP, CAV1, STARD3, FKBP10, EGF, F2, SERPINE1, CNGA3*), age, gender, grade and tumor stage	predict the 1-year and 3-year survival statuses of stomach adenocarcinoma patients	NR	H-L test: good	None	None
Xu et al. (109)	896	clinicopathological characteristics and laboratory parameters	log odds of positive lymph nodes, tumor size and lymphocyte-to-monocyte ratio	predict the prognosis of GC patients with triple-negative tumor markers	the AUCs for 1-, 3- and 5-year predictions were 0.870, 0.880 and 0.862	H-L test: good	the AUCs at 1, 3 and 5 years were 0.945, 0.845 and 0.896, respectively	None
Zhou et al. (110)	372	laboratory parameters and clinicopathological characteristics	serum ferritin (SF), sarcopenia, TNM stage system, and neoadjuvant chemotherapy	predict long-term survival for GC undergoing radical gastrectomy	The AUC of the nomogram for predicting the 3-year OS was 0.81,	NR	0.791*	None
Zhang et al. (111)	926	laboratory parameters and clinicopathological characteristics	pTNM stage, Borrmann tumor stage, and tumor marker index (TMI)	predict the prognosis of patients with AGC undergoing radical surgery	The AUCs of the nomogram for forecasting 3-year and 5-year OS rates were 0.791 and 0.767	H-L test: good	0.777* and 0.755*	None
Sun et al. (112)	1,013	demographic data and clinicopathological characteristics	age, histological grade, immunotherapy cycles and line of first immunotherapy	predict survival duration for patients with GC receiving immunotherapy	0.64#	H-L test: good	0.67#	NR
Ma et al. (113)	1,100	demographic data, laboratory parameters and clinicopathological characteristics	age, SRC ratio, tumor location, pT, pN, neoadjuvant chemotherapy, postoperative chemotherapy, neural invasion, preoperative CEA, and preoperative CA50	assess the overall survival (OS) of advanced gastric signet-ring cell carcinoma (GSRCC) patients after radical gastrectomy	the 1-, 3-, and 5-year AUCs of the nomogram in predicting advanced GSRCC were 0.791, 0.746, and 0.755, respectively	H-L test: good	None	None
Zhang et al. (114)	980	clinicopathological characteristics	tumor location, differentiation grade, N stage, chemotherapy, and number of regional nodes examined	predict CSS in middle-aged patients with EGC	0.749#	H-L test: good	0.744#	0.807#
He et al. (115)	3,492	laboratory parameters and clinicopathological characteristics	metastatic lymph node ratio (MLNR), age at surgery, type of gastrectomy, tumor size, T stage, and pathological grade	predict overall survival for gastric adenocarcinoma patients with radical gastrectomy	0.736#	H-L test: good	NR	0.712#

H-L test, Hosmer-Lemeshow test; NR, not reported; *area under the receiver operating characteristic curve, AUC; #the C-index.

## 4 Summary and prospect

CPMs, as important tools for evaluating the risk and prognosis of disease, have been widely used in the field of oncology. As an intuitive prediction tool, nomogram comprehensively take into account individual differences, thereby significantly enhancing the early identification rate of the high-risk GC population. They can optimize therapeutic strategies and provide patients with personalized prognostic information, while concurrently offering robust support for the decision-making process of clinical physicians. Despite remarkable progress in research and clinical application in the field of GC, nomogram still faces some challenges and limitations. Firstly, the data sources for most nomogram models are from single centers, and mainly undergo internal validation, lacking external validation from multi-center and large sample cohorts. Secondly, most models are constructed based on retrospective data, which may be the risk of bias. In addition, some nomograms rely on features that are relatively difficult to obtain, such as gene expression or sequencing data, as variables. This significantly restricts their clinical applicability.

Looking forward to the future, with the continuous progress of science and technology and deepening research, the application of nomogram in GC will become more extensive and accurate. Subsequent studies can further integrate multivariate data such as imaging techniques, biomarkers, and genetic information to optimize nomograms to improve prediction accuracy and clinical practicability. At the same time, larger sample sizes and multi-center studies are necessary to enhance external validation of nomograms, ensuring their applicability and accuracy across different patient populations. Moreover, combined with AI technologies such as machine learning and deep learning, the accuracy of nomogram models and the ability to extract complex features can be further improved, providing more scientific basis for personalized diagnosis and treatment of GC patients. Future studies should also incorporate patients’ lifestyle and environmental factors to construct more comprehensive predictive models. Additionally, developing dynamic web applications and mobile medical tools to enhance the usability of nomograms for clinicians and patients is also an important direction for future work. In summary, the application of nomograms in the field of GC will continue to provide important references and theoretical basis for clinical practice, and make greater contributions to improving the diagnosis, treatment, prognosis and quality of life of patients.
